# Transcranial Direct Current Stimulation of the Medial Prefrontal Cortex Has No Specific Effect on Self-referential Processes

**DOI:** 10.3389/fnhum.2020.00056

**Published:** 2020-03-11

**Authors:** Verena Mainz, Sara Britz, Saskia Doreen Forster, Barbara Drüke, Siegfried Gauggel

**Affiliations:** Institute of Medical Psychology and Medical Sociology, University Hospital of the RWTH Aachen University, Aachen, Germany

**Keywords:** transcranial direct current stimulation, medial prefrontal cortex, information processing, self-serving bias, menmic neglect effect

## Abstract

The processing of self-referential information can be influenced by transcranial magnetic stimulation (TMS). The present randomized controlled study investigated whether similar effects can be elicited through the application of transcranial direct current stimulation (tDCS) regarding the “self-serving bias” (SSB) and the “mnemic neglect effect” (MNE). Seventy-five healthy males (*M*_age_ = 25; SD = 4.3) were investigated in a between-groups design with random assignment by applying anodal, cathodal, or sham tDCS to the medial prefrontal cortex (mPFC). After stimulation, the participants judged if 80 personality traits (40 positive, 40 negative) were self-descriptive or not. Finally, the participants had to recall the previously presented adjectives. All three stimulation groups showed the expected SSB and MNE. Still, and contrary to our hypotheses, tDCS revealed neither a significant interaction effect between groups and valence concerning the number of chosen self-referential traits (*F*_(2,72)_ = 1.36, *p* = 0.26, $\eta_{\text{G}}^{2}$ = 0.02) nor an interaction effect between groups, valence, and self-reference concerning the percentage of recalled words (*F*_(2,71)_ = 0.69, *p* = 0.50, $\eta_{\text{G}}^{2}$ = 0.01). However, a *post hoc* inspection of effect sizes revealed that less negative traits were indicated as self-referential in the anodal compared to the cathodal group (ES: −0.59; CI: −1.16 to −0.03). Moreover, the participants showed—regardless of self-reference and type of stimulation—a better recall with tDCS in comparison to sham stimulation. Our results indicate that tDCS of the mPFC in healthy young men has no influence on the SSB and the MNE. However, tDCS seems to improve memory performance.

## Introduction

The “self ” is a relatively vaguely defined concept subsuming perception and knowledge about one’s self as a person such as knowledge about one’s personal characteristics, preferences, emotions, and behavior. The so-called “self-serving bias” (SSB) is a well-known phenomenon in social and personality psychology (Duval and Silvia, 2002; Dunning et al., 2004; Shepperd et al., 2008). Experimental studies show a SSB during the ascription of personality traits in healthy participants, that is, people refer more positive than negative characteristics to themselves and respond faster to positive as compared to negative self-referential traits (Alicke, 1985; Luber et al., 2012; Forster et al., 2019). Moreover, studies report the “mnemic neglect effect” (MNE), which comprises that people remember more positive as compared to negative self-referential traits (Sedikides and Green, 2009; Pinter et al., 2011).

These self-referential biases have attracted the attention of cognitive neuroscientists who want to identify the neural systems underlying the processing of self-relevant information. On a neuronal level, functional neuroimaging studies have identified a central role of the medial prefrontal cortex (mPFC) in self-processing. In a meta-analysis, Qin and Northoff (2011) found that neural activity in the cortical midline regions is self-specific and related to resting state activity. This meta-analysis has likewise reported an important and “unspecific” role for mPFC and posterior cingulate cortex in self-referential processing in the sense that these regions were recruited during the processing of self-specific (e.g., own name) and familiar stimuli (i.e., stimuli from personally known people) in comparison to non-self or non-familiar stimuli. Qin and Northoff (2011) suggest that the mPFC enables a “meta-representation” of stimuli required during judgments about one’s own traits.

Philippi et al. (2012) demonstrated the critical role of the mPFC for self-related processes using a human lesion approach. They found that the lesions to the mPFC were accompanied by a dysfunctional self-processing ability to the extent that the typical memory advantage conferred by self-related processing was absent in the patients. These findings were supported by two studies using transcranial magnetic stimulation (TMS). TMS-induced virtual lesions seem to influence self-evaluative processing (Kwan et al., 2007; Luber et al., 2012). Kwan et al. (2007) sought to assess the neural correlates of self-enhancement by applying TMS to the mPFC, the supplementary motor area (SMA), and the precuneus. They used the classical trait adjective judgment tasks with self and other judgments while single TMS pulses were delivered in a virtual lesion manner. Stimulation of the mPFC significantly reduced the overly positive self-perceptions, whereas stimulation of the SMA and the precuneus did not reveal similar effects. Very similar results were presented in a study by Luber et al. (2012) with single-pulse TMS to the mPFC and the left and the right parietal cortex. An interesting additional finding in both studies was that reaction times (RTs) were largely unaffected by TMS. The participants responded faster to positive and self-relevant traits as compared to the responses concerning another person. Nevertheless, both studies demonstrated that TMS to the mPFC decreased the participants’ tendency to self-enhance when compared to sham and/or different brain areas of stimulation. It is assumed that the stimulation leads to a disruption of a network that is responsible for self-referential information processing.

In the present study, we aimed to reproduce similar effects using a different brain stimulation method, namely, the transcranial direct current stimulation (tDCS). tDCS induces changes in the activity of the stimulated brain area by a direct current of low-level intensity (Nitsche and Paulus, 2000; Stagg and Nitsche, 2011). The orientation of the electric field (i.e., electrode position and polarity) determines the neuronal population that is stimulated. Current flows from the negatively charged cathode to the positively charged anode. It has been shown that surface anodal stimulation enhances, whereas surface cathodal stimulation reduces, the activity of superficial cortical neurons (Nitsche and Paulus, 2000). Assuming that the mPFC is functionally the core of self-specific semantic encoding, one should expect that decreasing the neuronal activity leads to a correspondent decrease in the tendency to self-enhance. Conversely, increasing the neuronal activity should lead to an increment of the self-enhancement tendency. Therefore, we speculate that tDCS of the cortical activity within the mPFC produces an excitatory or inhibitory effect (depending on the type of stimulation) that increases or decreases the functionality of self-specific semantic encoding. The potential of tDCS to also enhance positive self-referential processes has important treatment implications in mental disorders associated with poor self-image (as for, e.g., in patients with depression). We assume that an anodal stimulation of the mPFC could increase the tendency to self-enhance when compared to sham stimulation. Moreover, using cathodal stimulation, we expect the opposite effect, namely, a decreased tendency to self-enhance as compared to sham stimulation.

More specifically, we hypothesize to find the “typical” SSB effect during sham stimulation, which means that the participants choose more positive traits to be self-referential as compared to negative traits. During anodal as compared to sham stimulation, we expect to find an enhanced SSB due to its excitatory effect, i.e., the participants should indicate even more positive and less negative traits as self-referential. Cathodal stimulation should, due to its inhibitory effect, lead to a decreased SSB as compared to sham stimulation. Here the participants should indicate proportionally fewer positive and more negative traits as self-referential. RTs should stay largely unaffected by tDCS in all conditions, with the shortest RTs for chosen self-referential positive traits and longest RTs for chosen self-referential negative traits. Furthermore, we assume to find a “typical” memory bias, the MNE, during sham stimulation. The participants should remember more positive traits as compared to negative traits that were indicated as self-referential as compared to those indicated as non-self-referential. The positive and the negative traits that were indicated as non-self-referential should not differ with respect to memory recall. Again, anodal stimulation should lead to an increased MNE. The participants should recall more positive and less negative self-referential traits, whereas cathodal stimulation should minimize the MNE, indicated by a decreased recall of positive self-referential traits as compared to negative self-referential traits.

## Materials and Methods

### Participants and Procedure

Seventy-five male native German-speaking participants (*M*_age_ = 25; SD = 4.3, range 18–39) with no history of mental or neurological disease and no metallic implants near the head were recruited for the study *via* a flyer and word of mouth. The above inclusion criteria as well as the educational background were screened using a self-report questionnaire. All of the participants received verbal and written explanations of the purpose and procedures of the study, and written informed consent was obtained from all individual participants included in the study. Subsequently, the participants were randomly assigned to one of the three different tDCS conditions (anodal, cathodal, and sham; see Supplementary Table S1 Sociodemographic Data and Matching). All of the participants were tested in a single session consisting of a 20-min tDCS, followed by an experimental computer task. The participants were paid for their participation. The study was approved by the local Ethics Committee (EK161/15) in accordance with the Declaration of Helsinki.

### Transcranial Direct Current Stimulation

For tDCS, we used a DC-STIMULATOR by NeuroConn GmbH, Ilmenau, Germany. For all stimulation types (anodal, cathodal, or sham), a pair of rectangular rubber electrodes (5 cm × 7 cm; 35 cm^2^) covered in sponge pads was used. The type of stimulation is indistinguishable to the participants. Before placing the electrodes, the skin was cleaned with an alcohol spray solution. The electrodes’ sponge pads were coated with sodium chloride solution (NaCl, 0.9%) and then the electrodes were applied to the participant’s scalp at a current density of 0.057 μA/cm^2^. tDCS electrodes were placed according to the international 10-20 system (Jasper, 1958). To stimulate the mPFC, one electrode was placed horizontally 10% over the Nasion (Fpz) and the second horizontally 5% over the Inion (between Inion and Oz) to maximize the distance between both electrodes and thereby to decrease current shunted through the head and to increase current density in depth (Miranda et al., 2006).

To investigate anodal vs. cathodal stimulation, the polarity of the frontal electrode was switched, while the placement of the electrodes was identical for sham and real stimulation. To stimulate (anodal and cathodal), the current was faded in over the first 30 s, followed by a constant current of 2 mA (current density: 0.057 mA/cm^2^) applied for a duration of 20 min and 30 s of fading out. The impedance below 20 kΩ was automatically controlled by the stimulator device. We used the current intensity stimulation of 2 mA because the studies comparing dosage levels have demonstrated performance modulation on mPFC with 2 mA, but not with 1 mA (Boggio et al., 2006; Teo et al., 2011; Moos et al., 2012) or 0.6 mA (Clark et al., 2012). Concerning safety, tDCS studies have mostly adapted the use of relatively large electrodes (size nominally 25–35 cm^2^) and currents of 1–2 mA applied for durations of up to 20 min. This kind of current and tDCS procedure complies with the safety standards in humans (Nitsche et al., 2003; Iyer et al., 2005).

Sham stimulation starts with 8 s of fade in, followed by 30 s of direct current (2 mA) and 5 s of fade out to give the participants the same kind of skin sensation that occurs during normal stimulation. For the remaining session time and just for impedance control, only small current pulses occur (every 550 ms, 110 μA over 15 ms) instead of the stimulation current. Debriefing indicated that the participants were not able to identify neither stimulation nor sham condition.

### Self-referential Task

To assess the SSB and the MNE, 80 personality trait adjectives (40 adjectives classified as “positive” and 40 classified as “negative”) were chosen from the “Berliner Affektive Wort Liste—Reloaded” (BAWL-R; Võ et al., 2009). The influence of several stimuli properties on choice and memory was controlled for (Diependaele et al., 2012; see Supplementary Table S2 Matching of Stimuli Properties). The task was performed using Presentation^®^ software (Version 18.0, Neurobehavioral Systems Inc., Berkeley, CA, USA^1^) on a 23-inch computer screen.

#### Self-serving Bias

In the first part of the task, 100 adjectives were randomly presented in white color on a black screen for 2,500 ms each, intermitted by a variable fixation cross (between 500 and 800 ms). To negate the influence of primacy and recency effects, the participants performed 10 practice trials immediately before and after the set of 80 test trials. These trials were excluded from further analyses. The participants were asked to judge if each presented adjective was either self-descriptive or not self-descriptive by pressing one of two buttons (yes–no) on a keyboard with either the index or middle finger. The order of fingers applying for the answer (yes–no) was randomized across the participants. The dependent variables were the number of chosen positive and negative adjectives as self-referential and the RTs, respectively.

#### Mnemic Neglect Effect

Immediately after the completion of the first part, the participants were instructed to orally recall, in any order, as many of the previously presented adjectives as they could remember, irrespective of their choice of self-reference. The answers were voice-recorded and the participants were given 5 min to complete the task. The dependent variables were the percentage of recalled positive and negative self-referential and non-self-referential adjectives.

### Data Analyses

All data were analyzed using R x64 3.4.2 (R Core Team, 2017). Generalized eta-squared ($\eta_{\text{G}}^{2}$) effect sizes (Olejnik and Algina, 2003), recommended for repeated-measures analyses by Bakeman (2005), are reported for the ANOVA results regarding both the SSB and the MNE. To further analyze the effects of stimulation and due to the small sample sizes, the standardized effect sizes (ES) with the respective confidence intervals (CI, Hedges bias-corrected) are reported (Hedges and Olkin, 1985). The effect sizes greater than 0.50 were considered as relevant and are discussed in detail.

#### Self-serving Bias

Two 3 × 2 factorial repeated-measures analyses of variance with stimulation group (anodal, cathodal, and sham) as between-groups factor and valence of the adjectives (positive and negative) as within-group factor were calculated. The number of chosen self-referential positive and negative adjectives and the RTs served as the dependent variables, respectively. Please note that data from one participant in the cathodal group could not be included in the analysis of RTs due to an empty cell resulting from not indicating any of the negative adjectives as self-referential. Therefore, this analysis includes only 24 participants.

#### Mnemic Neglect Effect

A 3 × 2 × 2 factorial repeated-measures ANOVA with stimulation group (anodal, cathodal, and sham) as between-groups and valence of the adjectives (positive and negative) and self-reference (self-referential and non-self-referential) as within-group factors was calculated. Here the third factor (i.e., self-reference) emerges from the participants’ evaluation of the adjectives as self-referential or non-self-referential in the first part of the task. The dependent variables were the percentage of recalled self-referential, non-self-referential, positive, and negative adjectives. We chose to report the percentages instead of the absolute numbers of the recalled adjectives to account for the different amounts of misses during the judgment task between the participants.

## Results

### Self-serving Bias

**Figure 1:**
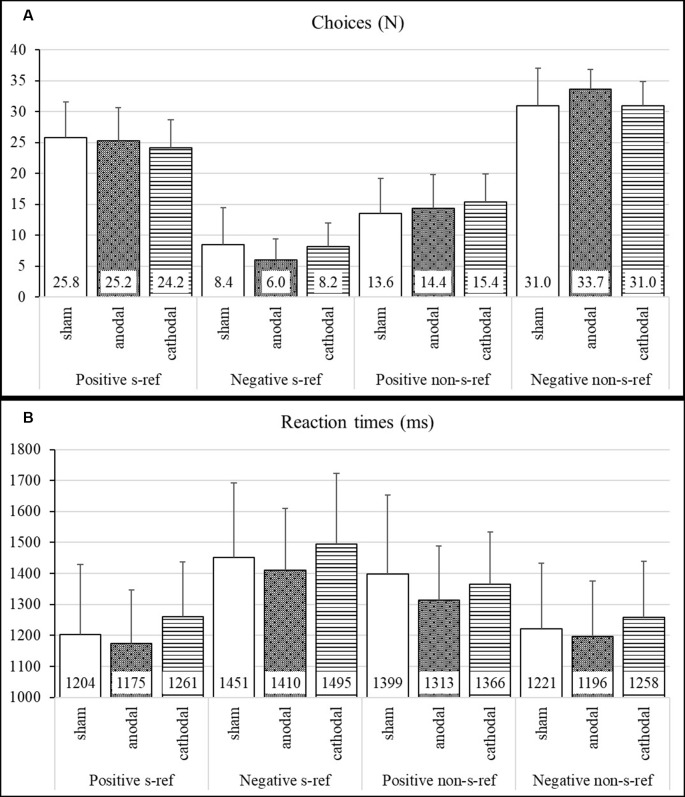
Self-serving bias—number of chosen adjectives and reaction times (RTs) in the stimulation groups. **(A)** Group means and standard deviations for the number of choices (*N*). **(B)** Group means and standard deviations for RTs in milliseconds (ms).

Means, standard deviations, standard error of means, and effect sizes of the participants’ task performance (RTs and number of chosen self-referential and non-self-referential adjectives) are shown in Figure 1 and Table 1. The ANOVA on choice of self-referential adjectives revealed a significant main effect for valence (*F*_(1,72)_ = 472.8, *p* < 0.001, $\eta_{\text{G}}^{2}$ = 0.77) and neither a significant main effect for stimulation group (*F*_(2,72)_ = 1.15, *p* = 0.32, $\eta_{\text{G}}^{2}$ = 0.02) nor an interaction effect between valence and stimulation group (*F*_(2,72)_ = 1.36, *p* = 0.26, $\eta_{\text{G}}^{2}$ = 0.02). The medium effect size showed that the participants of the anodal stimulation group indicated less negative adjectives as self-referential compared to the cathodal stimulation group (ES: = −0.59; CI: −1.16 to −0.03).

**Table 1:** The number of chosen adjectives and reaction times in the stimulation groups.

Choices^b^						
Variable^a^	Group (*N*)	M	SD	SEM	ES (CI)^c^ Anodal	ES (CI)^c^ Cathodal
pos s-ref	Sham (25)	25.8	5.8	1.2	0.11 (−0.45; 0.66)	0.30 (−0.25; 0.86)
	Anodal (25)	25.2	5.4	1.1		0.20 (−0.36; 0.75)
	Cathodal (25)	24.2	4.5	0.9		
neg s-ref	Sham (25)	8.4	6.0	1.2	0.48 (−0.08; 1.04)	0.04 (−0.52; 0.59)
	Anodal (25)	6.0	3.5	0.7		**−0.59** (−1.16; −0.03)
	Cathodal (25)	8.2	3.8	0.8		
pos n-s-ref	Sham (25)	13.6	5.7	1.1	−0.14 (−0.70; 0.41)	−0.35 (−0.90; 0.21)
	Anodal (25)	14.4	5.4	1.1		−0.20 (−0.75; 0.36)
	Cathodal (25)	15.4	4.5	0.9		
neg n-s-ref	Sham (25)	31.0	6.1	1.1	**−0.55** (−1.11; 0.02)	0.00 (−0.55; 0.55)
	Anodal (25)	33.7	3.2	0.6		**0.75** (0.17; 1.32)
	Cathodal (25)	31.0	3.9	0.8		
**Reaction times^d^**						
pos s-ref	Sham (25)	1,204	225	45.0	0.14 (−0.41;.70)	−0.28 (−0.84; 0.29)
	Anodal (25)	1,175	171	34.1		−0.49 (−1.05; 0.08)
	Cathodal (24)	1,261	177	36.0		
neg s-ref	Sham (25)	1,451	242	48.3	0.18 (−0.37; 0.74)	−0.18 (−0.75; 0.38)
	Anodal (25)	1,410	200	40.0		−0.39 (−0.96; 0.17)
	Cathodal (24)	1,495	227	46.4		
pos n-s-ref	Sham (25)	1,399	255	51.0	0.39 (−0.17; 0.95)	0.15 (−0.41; 0.71)
	Anodal (25)	1,313	176	35.2		−0.30 (−0.87; 0.26)
	Cathodal (24)	1,366	167	34.1		
neg n-s-ref	Sham (25)	1,221	213	42.7	0.13 (−0.43; 0.68)	−0.18 (−0.75; 0.38)
	Anodal (25)	1,196	179	35.7		−0.34 (−0.90; 0.23)
	Cathodal (24)	1,258	181	36.9		

*^a^Type of adjectives: pos s-ref, positive self-referential; neg s-ref, negative self-referential; pos n-s-ref, positive non-self-referential; neg n-s-ref, negative non-self-referential; ^b^Choices, number of chosen adjectives; ^c^ES (CI), standardized effect sizes and confidence intervals (95%); ES >0.50 are indicated in bold; ^d^Reaction times indicated in milliseconds*.

The ANOVA on RTs for self-referential adjectives revealed a significant main effect for valence (*F*_(1,71)_ = 120.25, *p* < 0.001, $\eta_{\text{G}}^{2}$ = 0.25) and neither a significant main effect for stimulation group (*F*_(2,71)_ = 1.29, *p* = 0.28, $\eta_{\text{G}}^{2}$ = 0.03) nor an interaction (*F*_(2,71)_ = 0.04, *p* = 0.96, $\eta_{\text{G}}^{2}$ = 0.00).

### Mnemic Neglect Effect

**Figure 2:**
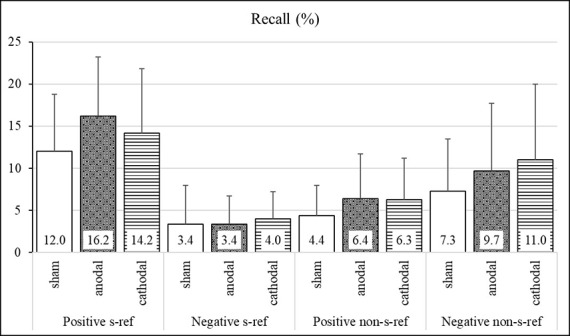
Mnemic neglect effect—percentage of recalled adjectives in the stimulation groups. Group means and standard deviations of the percentage of recalled adjectives (%).

The means, standard deviations, standard error of means, and effect sizes of the participants’ task performance are shown in Figure 2 and Table 2. The ANOVA with percentage of recalled self-referential and non-self-referential positive and negative adjectives revealed significant main effects for valence (*F*_(1,71)_ = 54.99, *p* < 0.001, $\eta_{\text{G}}^{2}$ = 0.08) and self-reference (*F*_(1,71)_ = 4.44, *p* < 0.05, $\eta_{\text{G}}^{2}$ = 0.01) and a significant interaction effect between valence and self-reference (*F*_(1,71)_ = 74.17, *p* < 0.001, $\eta_{\text{G}}^{2}$ = 0.26). Neither the main effect for the stimulation group (*F*_(2,71)_ = 3.04, *p* = 0.05, $\eta_{\text{G}}^{2}$ = 0.03) nor the interactions between the stimulation group and the valence (*F*_(2,71)_ = 1.82, *p* = 0.17, $\eta_{\text{G}}^{2}$ = 0.01), stimulation group and self-reference (*F*_(2,71)_ = 0.4, *p* = 0.63, $\eta_{\text{G}}^{2}$ = 0.00), and stimulation group, valence, and self-reference (*F*_(2,71)_ = 0.69, *p* = 0.50, $\eta_{\text{G}}^{2}$ = 0.01) reached significance. The medium effect size revealed that the participants of the anodal stimulation group recalled significantly more positive adjectives that were indicated as self-referential as compared to the participants of the sham stimulation group (ES: −0.60; CI: −1.16 to −0.03).

**Table 2:** Percentage of the recalled adjectives in the stimulation groups.

Recall^b^						
Variables^a^	Group (*N*)	M	SD	SEM	ES (CI)^c^ Anodal	ES (CI)^c^ Cathodal
pos s-ref	Sham (25)	12.0	6.8	1.4	**−0.60** (−1.16; −0.03)	−0.30 (−0.86; 0.26)
	Anodal (25)	16.2	7.0	1.4		0.27 (−0.29; 0.83)
	Cathodal (24)	14.2	7.6	1.6		
neg s-ref	Sham (25)	3.4	4.6	0.9	0.00 (−0.55; 0.55)	−0.15 (−0.71; 0.41)
	Anodal (25)	3.4	3.3	0.7		−0.18 (−0.74; 0.38)
	Cathodal (24)	4.0	3.2	0.6		
pos n-s-ref	Sham (25)	4.4	3.6	0.7	−0.43 (−1.0; 0.13)	−0.44 (−1.0; 0.13)
	Anodal (25)	6.4	5.3	1.1		0.02 (−0.54; 0.58)
	Cathodal (24)	6.3	4.9	1.1		
neg n-s-ref	Sham (25)	7.3	6.2	1.3	−0.33 (−0.89; 0.23)	−0.47 (−1.04; 0.10)
	Anodal (25)	9.7	8.0	1.6		−0.15 (−0.71; 0.41)
	Cathodal (24)	11.0	9.0	1.8		
pos total	Sham (25)	16.4	7.8	1.6	**−0.79** (−1.37; −0.21)	**−0.52** (−1.09; 0.05)
	Anodal (25)	22.5	7.4	1.5		0.24 (−0.32; 0.80)
	Cathodal (24)	20.6	8.2	1.7		
neg total	Sham (25)	10.6	7.5	1.5	−0.30 (−0.86; 0.26)	−0.52 (−1.09; 0.05)
	Anodal (25)	13.1	8.8	1.8		−0.21 (−0.77; 0.35)
	Cathodal (24)	15.0	9.1	1.9		

*^a^Type of adjectives: pos s-ref, positive self-referential; neg s-ref, negative self-referential; pos n-s-ref, positive non-self-referential; neg n-s-ref, negative non-self-referential; ^b^Recall, percentage of recalled adjectives; ^c^ES (CI), standardized effect sizes and confidence intervals (95%); ES >0.50 are indicated in bold*.

Table 2 additionally shows the effect sizes between the stimulation groups with regard to the percentage of recalled words from the total of positive and negative adjectives, irrespective of the self-reference. The results reveal that the participants of the anodal stimulation group recalled more adjectives from the total of positive adjectives as compared to the participants of the sham stimulation group (ES: −0.79; CI: −1.37 to −0.21). The cathodal stimulation led to a recall of more positive (ES: −0.52; CI: −1.09 to 0.05) and negative (ES: −0.52; CI: −1.09 to 0.05) adjectives from the total of adjectives as compared to that of the participants of the sham stimulation group.

## Discussion

So far, to the best of our knowledge, only two studies demonstrated the causal role of certain brain regions in self-referential processing, while literature depicts the mPFC to functionally play the central role in self-specific encoding (Qin and Northoff, 2011). In the two studies, it was shown that TMS of the mPFC led to a disruption of self-referential processing (Kwan et al., 2007; Luber et al., 2012). Applying TMS and therewith causing a decreasing neuronal activity within the mPFC revealed a correspondent diminished tendency to self-enhance in comparison to a sham condition or the stimulation of other brain areas, while RTs remained largely unaffected. The aim of the present study was to investigate in how far tDCS to the mPFC similarly affects self-referential information processing. Specifically, we investigated the differential effects of anodal, cathodal, and sham tDCS with regard to the SSB and the MNE.

Concerning the SSB, many studies have shown that people refer more positive than negative characteristics to themselves and respond faster to positive as compared to negative self-referential traits (Luber et al., 2012; Forster et al., 2019). Our results revealed the typical SSB pattern in all three stimulation conditions. The participants indicated more positive than negative adjectives to be self-referential, with the fastest RT for self-referential positive decisions. However, analyses did not reveal the hypothesized findings of an overall enhanced or diminished SSB as expected through the application of an excitatory or inhibitory tDCS. An inspection of effect sizes (Table 1) showed that the anodal stimulation influenced choice, leading to the selection of more negative adjectives as non-self-referential as compared to both cathodal and sham condition. The cathodal stimulation revealed a decreased tendency to self-enhance as compared to the anodal condition, which is indicated by a selection of more negative adjectives as self-referential. However, due to non-significant main and interaction effects, these findings require replication and confirmation.

The MNE refers to a memory advantage during recall tasks for positive compared to negative information assigned to one’s own person (Sedikides and Green, 2009; Sedikides et al., 2016). Overall the results reveal the typical MNE within the three conditions. There was a significant recall advantage for positive self-referential in comparison to non-self-referential adjectives and, more importantly, for positive in comparison to negative self-referential adjectives, which represents the MNE within the groups. However, our analyses did not reveal any differences between the stimulation conditions. Consequently, we could not corroborate our hypotheses of an increased or minimized MNE as a consequence of an anodal or cathodal stimulation, respectively. However, we found a weak evidence of a medium effect size, indicating a recall advantage for positive self-referential adjectives in the anodal stimulation group as compared to the sham stimulation group. This speaks in favor of a self-enhancing bias tendency. In an additional inspection of effect sizes, looking at the recall advantage of overall positive and negative adjectives between groups, a recall advantage for overall positive adjectives became evident in the anodal and the cathodal stimulations in comparison to the sham condition. Moreover, the cathodal stimulation group also recalled more negative adjectives as compared to the sham group. This difference speaks in favor of a rather general than valence-specific recall advantage through stimulation.

We want to point out several study limitations and possible implications for future work. First, in the two TMS studies, male and female participants were investigated. Although it has been shown that the self-serving biases are robust and transversal to age, gender, and culture (Symons and Johnson, 1997; Sedikides et al., 2003), we decided to investigate only male participants. With this selection, we wanted to keep the results homogeneous with respect to possible gender effects. However, we cannot rule out that tDCS has a different effect on female participants (see e.g., Lee et al., 2018). Future studies should systematically investigate and include the role of gender and tDCS in self-serving biases.

Second, in both TMS studies, the mPFC and other cortical areas (e.g., parietal cortex) were stimulated. The results revealed a reduced self-serving tendency through TMS of the mPFC and, to a lesser degree, through stimulation of the precuneus and the posterior cortical regions (Kwan et al., 2007; Luber et al., 2012). Furthermore, not only the region of stimulation varied between the studies but also the time and the strength of stimulation. Luber et al. (2012) argued that a stronger stimulation had a more disruptive effect below the coil, affected a more extensive region, and reached more regions in depth. In comparison to the two TMS studies, we did not vary the regions nor the duration or the strength of stimulation. More generally, it has to be discussed in how far the tDCS differs from TMS methodologically and in its effectiveness. In contrast to TMS, which produces action potentials in neural membranes, tDCS can be understood as a neuromodulatory intervention which causes a (de- or hyper-) polarization of the exposed tissue. Its effects depend on the current density and the stimulation duration (Nitsche et al., 2008). While surface anodal stimulation typically has excitatory effects, cathodal stimulation reduces the activity of superficial cortical neurons (Nitsche and Paulus, 2000). Still the neurons situated in deeper cortical sulci can be oppositely affected and, because of the large electrode size, adjacent cortical areas might also be stimulated (Nitsche et al., 2008; Jacobson et al., 2012; Bellaïche et al., 2013). Therefore, it is possible that other results would be obtained with tDCS if other brain areas were stimulated with different stimulation strengths.

Third, there is an ongoing debate about TMS and the tDCS effects and their possible limits in healthy participants (Hoy et al., 2013). Although tDCS alters and may improve the cognitive performance in certain patient populations (Faehling and Plewnia, 2016), it may fail to enhance the performance in healthy populations due to ceiling effects (Bellaïche et al., 2013; Coffman et al., 2014; Horvath et al., 2015).

Fourth, Luber et al. (2012) found a disrupting effect in the “self” condition but no effect in the condition where a best friend was judged by the participant. In our study, we decided to focus on self-referential choices and valence as well as memory differences comparing self and non-self-choices. Therefore, we cannot rule out that the tDCS effects could have emerged when comparing self-ratings vs. other ratings instead of self-ratings and non-self-ratings only.

Finally, it has to be mentioned that our study was powered to detect large effects. A more detailed analysis of the results of the two TMS studies shows that a large effect size cannot be assumed without further ado. Luber et al. (2012, p. 7) report statistical trends, and Kwan et al. (2007, p. 383) report a non-significant three-way interaction. Our effect sizes indicate that repeating the present tDCS study with a larger sample capable of detecting weak effects may allow a more conclusive interpretation of the findings.

In summary, we cannot rule out that methodological problems (e.g., sample size) were responsible for the non-significant stimulation results. Divergent results, in comparison to the findings in the studies using TMS, can be explained by differences in the depth, the strength, and the region specificity of the interference signal caused by tDCS. Finally, it could be that tDCS fails to affect specific “higher-order” metacognitive processes concerning self-specific contents as investigated in our study (see also Schäfer and Frings, 2019). Rather, it has the potential to influence more basal processes like memory, attention, visual, and motor processes (Nitsche and Paulus, 2000; Jacobson et al., 2012; Moos et al., 2012; Frings et al., 2018). Although behaviorally we found typical self-serving biases in our samples, tDCS seems to have a rather unspecific neuronal effect with regard to the investigated tasks.

However, we believe that task performance and SSB involve complex cognitive processes including self-monitoring and meta-knowledge. To understand the definite causal link between neural processes and self-referential information processing therefore needs further investigations. In how far tDCS has the potential to exert a more specific effect in patients where processes are affected through symptopathology remains to be elucidated. Patients with affective disorders, especially those with major depression, are characterized by profound self-referential biases, and several studies have shown promising results concerning the effects of tDCS on symptopathology (see e.g., Brunoni et al., 2012). The advantages of tDCS as an easily applicable, low-cost stimulation with little to no adverse effects are undeniable arguments to perpetuate and extend the investigations of its effects in healthy but moreover in patient populations.

## Data Availability Statement

The datasets generated during and/or analyzed during the current study are available from the corresponding author on reasonable request.

## Ethics Statement

All procedures performed in studies involving human participants were in accordance with the ethical standards of the institutional and/or national research committee and with the 1964 Helsinki Declaration and its later amendments or comparable ethical standards.

## Author Contributions

VM, SG, and BD contributed in the conception and the design of the study. VM organized the database and wrote the first draft of the manuscript. VM, SB, and SF performed the statistical analysis. SG wrote sections of the manuscript. All of the authors contributed to the manuscript revision and read and approved the submitted version.

## Conflict of Interest

The authors declare that the research was conducted in the absence of any commercial or financial relationships that could be construed as a potential conflict of interest.

## Footnotes

^1^http://www.neurobs.com
