# Supplementary material for: Transcranial Direct Current Stimulation of the Medial Prefrontal Cortex Has No Specific Effect on Self-referential Processes
Source: Front Hum Neurosci. 2020 Mar 11;14:56. doi: 10.3389/fnhum.2020.00056 (PMC7078362; doi:10.3389/fnhum.2020.00056)
Supplement: Supplementary file 1 [file Data_Sheet_1.pdf]

Supplementary Material

Table S1 - Sociodemographic data of the three stimulation groups and matching of participants

Variable	Group	*N*	*M*	*SD*	$p_{cathodal*}$	p_{sham*}
**Age (years)**	Anodal	25	25.8	5.0	0.1	1.0
	Cathodal	25	23.7	2.8		0.1
	Sham	25	25.8	4.6		
**Years of education**	Anodal	25	16.3	2.2	0.9	0.8
	Cathodal	25	16.1	3.3		0.7
	Sham	25	16.5	4.0		

*Pairwise comparisons

As participants were randomly assigned to one of the three stimulation conditions (anodal, cathodal, sham) it was tested with a multivariate three-factorial analysis of variance (MANOVA) whether samples differed according to age (indicated in years) or education status (indicated in years of education). The MANOVA revealed no significant main effect ($F(4,144) = 1.25$, $p = .29$, $\eta p^2 = .03$). Means, standard deviations of age and years of education in the three groups and pairwise comparisons are displayed, and show, that neither age nor education significantly differed between the three groups.

Table S2 - Matching of the stimuli properties

Variable	Adjective list	*M*	*SD*	*F*	*p**	ηp^2
**valence**	negative	1.45	0.59	0.16	0.69	0.00
	positive	1.39	0.64			
**arousal**	negative	2.84	0.78	3.14	0.08	0.04
	positive	2.56	0.63			
**imageability**	negative	3.27	0.80	0.25	0.62	0.00
	positive	3.18	0.83			
**letter length**	negative	6.83	1.15	0.82	0.37	0.01
	positive	6.58	1.32			
**frequency**	negative	11.67	27.92	1.25	0.27	0.02
	positive	26.19	77.25			

*Post hoc univariate analyses of variance (ANOVA)

To match the adjective lists (40 positive and 40 negative personality trait adjectives) according to letter length, frequency of occurrence, emotionality, imageability, and arousal a multivariate two-factorial analysis of variance (MANOVA) was calculated with post hoc univariate analyses between positive and negative adjective lists to detect differences between the lists with respect to the above stimuli properties. The stimuli properties were taken from the BAWL-R (Vo et al., 2009). The MANOVA revealed no significant main effect ($F(5,74) = 1.15$, $p = .34$, $\eta p^2 = .07$). Means and standard deviations as well as post hoc univariate test results are displayed, and show, that stimuli properties of positive and negative adjective lists matched according to letter length, frequency of occurrence, emotionality, imageability, and arousal.
